# Prognostic value and therapeutic potential of NEK family in stomach adenocarcinoma

**DOI:** 10.7150/jca.90197

**Published:** 2024-04-15

**Authors:** Xunjian Zhou, Hui Nie, Chunrong Wang, Xiaoqian Yu, Xuejie Yang, Xiaoyun He, Chunlin Ou

**Affiliations:** 1 Department of Pathology, The First Hospital of Changsha (The Affiliated Changsha Hospital of Xiangya School of Medicine, Central South University), Changsha 410013, Hunan, China.; 2 Department of Pathology, Xiangya Hospital, Central South University, Changsha 410008, Hunan, China.; 3 Departments of Ultrasound Imaging, Xiangya Hospital, Central South University, Changsha 410008, Hunan, China.; 4 National Clinical Research Center for Geriatric Disorders, Xiangya Hospital, Central South University, Changsha 410008, Hunan, China.

**Keywords:** GATA family, stomach adenocarcinoma, prognosis, methylation, immune cells, biomarkers

## Abstract

Never in mitosis gene A-related kinase (NEK) is an 11-membered family of serine/threonine kinases (NEK1-NEK11), which are known to play important roles in the formation and development of cancer. However, few studies have examined the roles of these kinases in the development of stomach adenocarcinoma (STAD). In this study, we conducted a comprehensive analysis of the relationships between the NEKs family members and STAD. The differential expression of the *NEK* genes in STAD was validated using The Cancer Genome Atlas (TCGA) and Tumor Immune Estimation Resource (TIMER) databases, and their prognostic and diagnostic values of NEKs in STAD were assessed using the Kaplan-Meier plotter and TCGA data. The effect of NEK expression on immune cell infiltration in STAD was analysed using the TIMER and TISIDB databases. The expression levels of the majority of the NEK family members were consistently upregulated in STAD, whereas that of NEK10 was downregulated. The upregulation of NEK2/3/4/5/6/8 was closely associated with clinicopathological parameters of patients, and the overexpressed levels of these proteins had good diagnostic value for the disease. NEK1/8/9/10/11 expression correlated with poor overall survival and post-progressive survival, whereas a higher NEK1/6/9/11 level implied worse first progressive survival. Gene Ontology and Kyoto Encyclopedia of Genes and Genomes enrichment analyses revealed that the NEKs may be related to immunological responses. Additionally, our study confirmed that these kinases correlated with immune cell infiltration and different immune infiltration subtypes in STAD. Our results suggest that NEK9 in particular has the potential to be used as a diagnostic and prognostic biomarker of STAD development and progression and an immune target for treatment of the disease. These findings expand our understanding of the biological functions of the NEK family members in STAD.

## Introduction

Stomach adenocarcinoma (STAD) is a malignant disease that occurs in the cells of the stomach glands, and accounts for 95% of gastric cancers 1. Compared with other cancers, this disease has one of the highest morbidity and mortality rates 2, 3. The limited clinical features of early STAD render its timely diagnosis difficult, increasing the risk of progression to advanced gastric cancer 4. Although the popularisation of endoscopic technology, has resulted in breakthrough progress being made in the detection and treatment of early STAD, the long-term survival rate of patients with advanced disease is still low. To improve the prognosis of patients, current research should focus on identifying STAD-associated genes that can be used for the early detection of the disease or as therapeutic targets.

Never in mitosis gene A-related kinase (NEK) is a family of serine/threonine kinases made up of 11 members: NEK1-NEK11 5. These kinases contain serine/threonine residues at the activation modification sites within the activation loop 6. Most members of the NEK family play indispensable roles in eukaryotic mitosis and cell-cycle regulation 7. Consistent with the fact that the occurrence and development of cancer are inseparable from dysregulation of the cell cycle, an increasing number of studies have revealed a potential relationship between the NEK family and various cancer types 8, 9. For example, the level of NEK1 expression has been found to be abnormally elevated in human prostate cancer, making the protein a potential therapeutic target 10. Moreover, the expression of NEK2 is highly linked to cancer occurrence, development, and drug resistance 11. Additionally, NEK4 has been shown to play a key role in lung and colorectal cancers 12, 13. However, the roles of the different NEK family members in STAD have not been elucidated.

Currently, databases for tumor analysis include the Tumor Immune Estimation Resource (TIMER), the Cancer Genome Atlas (TCGA), Gene Expression Omnibus (GEO), Connectivity map (CMAP) 14, 16, Cancer Cell Line Encyclopedia (CCLE) 17, 19, etc. In this study, the potential role of NEK family members in STAD was explored through the analysis of some database data and experimental verification. To achieve this, data on the differential expression of the NEK genes in gastric cancer were obtained from various public databases and the correlations of the expression levels with clinicopathological characteristics, prognosis, signalling pathways, and immune infiltration were analysed.

## Materials and Methods

### The Cancer Genome Atlas (TCGA) HCC samples

TCGA, the most comprehensive cancer genome database available, contains clinical data from more than 11,000 patients across 33 cancer types 20. We retrieved the clinical data of patients with STAD from this database and analysed the expression of the NEKs in the disease. The results were visualised using the online tool of the Xiantao Academic Network (https://www.xiantao.love/products).

### Exclusion and inclusion criteria

Exclusion and inclusion criteria Eligible patients included in this article are in accordance with the following inclusion criteria: (1) pathological confirmation of the diagnosis; (2) prior to resection, none of the patients had received any type of therapy, including chemotherapy, radiation, or immunotherapy; (3) complete clinicopathological data. The detailed clinic parameters of enrolled patients were presented in **Supplementary Table 1**. Exclusion criteria included the following: (1) other treatments were used after the operation; (2) vital organ dysfunction; (3) other organ tumors.

### Differential expression analysis of the *NEK* genes in STAD

TIMER database is a comprehensive web resource that provides users with quick and intuitive access to data for intuitively analysing the relationship between different types of cancer and immune infiltration (https://cistrome.shinyapps.io/timer/) 21. By entering different modules, the correlation between gene expression, mutant genes, and immune infiltration levels can be retrieved. The differential expression of genes between different tumours and normal tissues can also be determined intuitively. Additionally, the site facilitates analysis of the correlations between genes 22.

### Analysis of the association between *NEK* expression and STAD clinicopathological characteristics

UALCAN (http://ualcan.path.uab.edu/index.html) provides users with a convenient way to analyse the relationship between gene expression and characteristics using TCGA data 23. Additionally, it can be used to analyse differential gene expression and methylation.

### Analysis of the prognostic value of *NEKs* in STAD

Kaplan-Meier plotter (http://kmplot.com) data were obtained from various data platforms, including Gene Expression Omnibus (GEO), European Genome-phenome Archive (EGA), and TCGA. Kaplan-Meier survival analysis was used to analyse the human survival data for 31 types of cancer, including those of the breast, stomach, liver, and lung cancers 24.

### Analysis of *NEK* gene alterations in STAD

The cBioPortal for Cancer Genomics (http://cbioportal.org) database integrates information on at least 30 cancer types, providing researchers with more intuitive access to epigenetic, gene expression, and proteomics data 25. We used this database to explore NEK gene alterations in STAD. MethSurv is a visual analysis tool for exploring methylation biomarkers associated with cancer patient survival (https://biit.cs.ut.ee/methsurv) 26-28. Gene Set Cancer Analysis (GSCA; https://guolab.wchscu.cn/GSCA/#/) data were used to analyse the relationship of promoter methylation levels in the NEK genes to patient survival differences in STAD 29. These data were also used to predict drug sensitivity.

### Analysis of *NEK* gene co-expression and enrichment in signalling pathways in STAD

Gene Expression Profiling Interactive Analysis (GEPIA), which integrates tumour and normal tissue samples from the Genotype-Tissue Expression (GTEx) and TCGA databases, is a platform for gene differential expression, survival, and correlation analyses (http://gepia2.cancer-pku.cn/) 30. STRING is the most comprehensive database for predicting and analysing protein interactions (https://string-db.org/) 31. Metascape is a robust functional annotation database for acquiring Gene Ontology (GO) and Kyoto Encyclopedia of Genes and Genomes (KEGG) enrichment pathway information on genes (https://metascape.org) 32.

### Analysis of the association of *NEK* expression with immune infiltration in STAD

TIMER, one of the most common online tools used to analyse immune cell infiltration in tumour tissue, mainly analyses the infiltration of six types of immune cells, (viz.B cells, CD4+ T cells, CD8+ T cells, neutrophils, macrophages, and dendritic cells) in tumours. The TISIDB website provides information on the interaction between tumours and immune cells (http://cis.hku.hk/TISIDB) 33. Using these databases, we fully explored the relationships between the NEKs, STAD, and immune cells to predict the influence of the kinases on disease occurrence and development and identify if any of them can be immunotherapeutic targets.

### Patients' tissue samples

In total, 22 pairs of matched adjacent normal tissue samples and paraffin-embedded archival STAD specimens were collected from Xiangya Hospital (Changsha, P. R. China). These clinical specimens were collected with the approval of the Research Ethics Committee of the Xiangya Hospital of the Central South University.

Patients from whom the tumour specimens were collected met the following inclusion criteria: (1) confirmed diagnosis by pathology; (2) no prior treatment, including chemotherapy, radiotherapy, or immunotherapy before resection; and (3) complete clinicopathological data. The exclusion criteria were patients who received other treatments after surgery, had vital organ dysfunction, and had tumours in other organs.

### Isolation of RNA from formalin-fixed paraffin-embedded samples

The formalin-fixed paraffin-embedded (FFPE) colon cancer or normal tissue samples were first deparaffinised with xylene. Then, total RNA was extracted from the cells using the Total RNA AmoyDx® FFPE RNA Extraction Kit (Cat. # 8.02.0019; AmoyDx, Xiamen, P. R. China).

### Quantitative real-time polymerase chain reaction

The RNAs isolated from the various tissue samples were amplified and subsequently evaluated using the quantitative real-time polymerase chain reaction (qRT-PCR). **Table 1** displays the qRT-PCR primer sequences.

### Statistical analysis

The T test was used for the differential expression analysis, whereas the Chi-squared test was used for the analysis of clinicopathological characteristics. Differences with a* P* value of < 0.05 were considered statistically significant. The selection conditions for gene co-expression were |Log_2_FC| > 1 and *P* < 0.05.

## Results

### Expression of the *NEK* genes in STAD and normal gastric tissues

**Figure 1:**
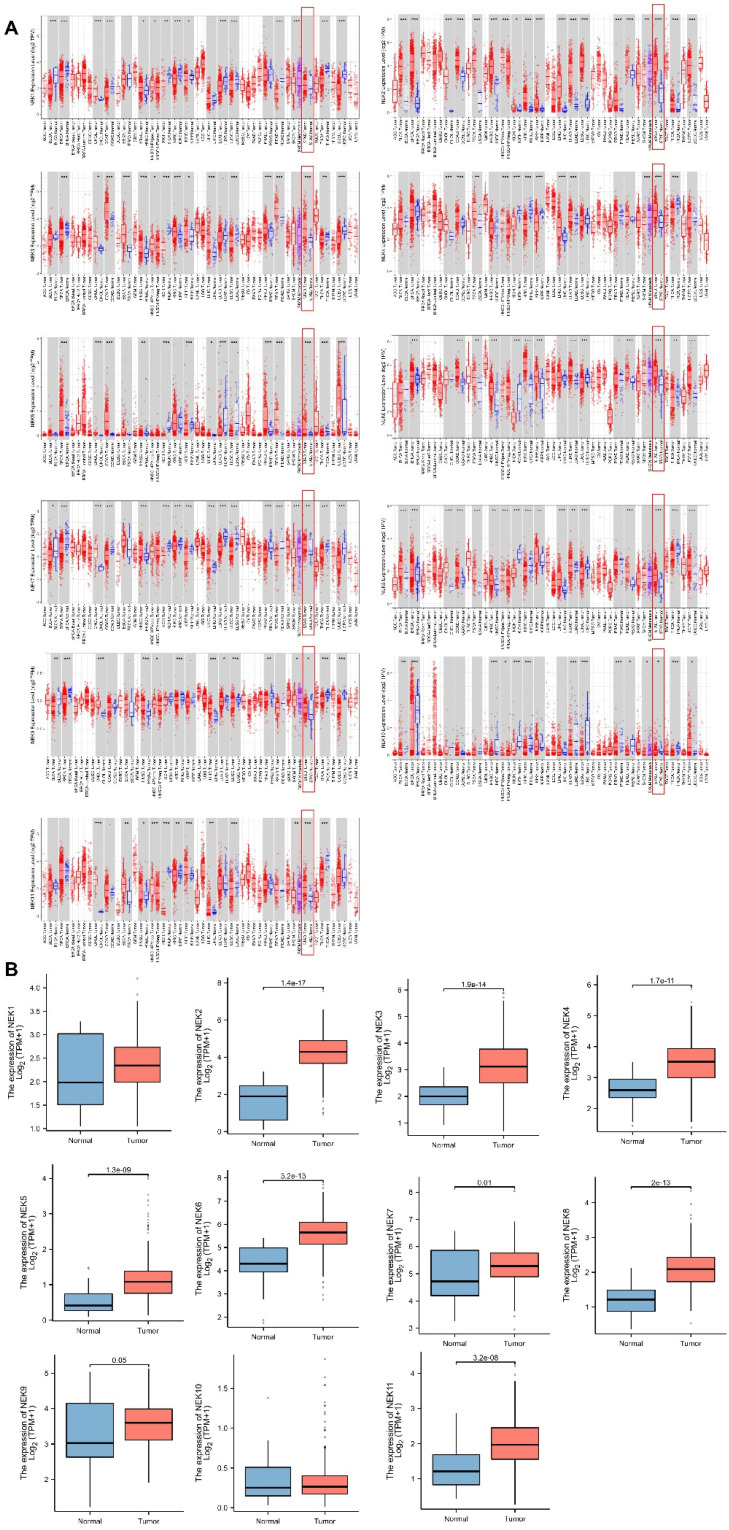
*NEK* family members are differentially expressed in STAD. (**A**) Expression levels of the *NEK* genes in different cancers compared with those in normal tissues (TIMER);.*P < 0.05, **P < 0.01, ***P < 0.01. (**B**) *NEK* gene expression levels in patients with STAD.

First, we conducted correlation studies on the expression of the *NEK* genes in STAD using the TIMER database. The results revealed that, except for *NEK10*, the expression levels of the other *NEK* genes were upregulated in the STAD tissues relative to the levels in the adjacent normal tissues (**Figure 1A**). Next, we verified the expression of these genes in STAD using TCGA data from the Xiantao Academic Network. As illustrated in** Figure 1B**, *NEK2, NEK3, NEK4, NEK5, NEK6, NEK7, NEK8, NEK9,* and *NEK11* were overexpressed in the STAD tissue, and the difference in expression levels between the two groups of tissue was statistically significant (*P* < 0.05). However, the NEK1 and NEK10 expression levels were not notably different between the STAD and normal gastric tissue samples.

### Association of NEK expression with clinicopathological characteristics

**Figure 2:**
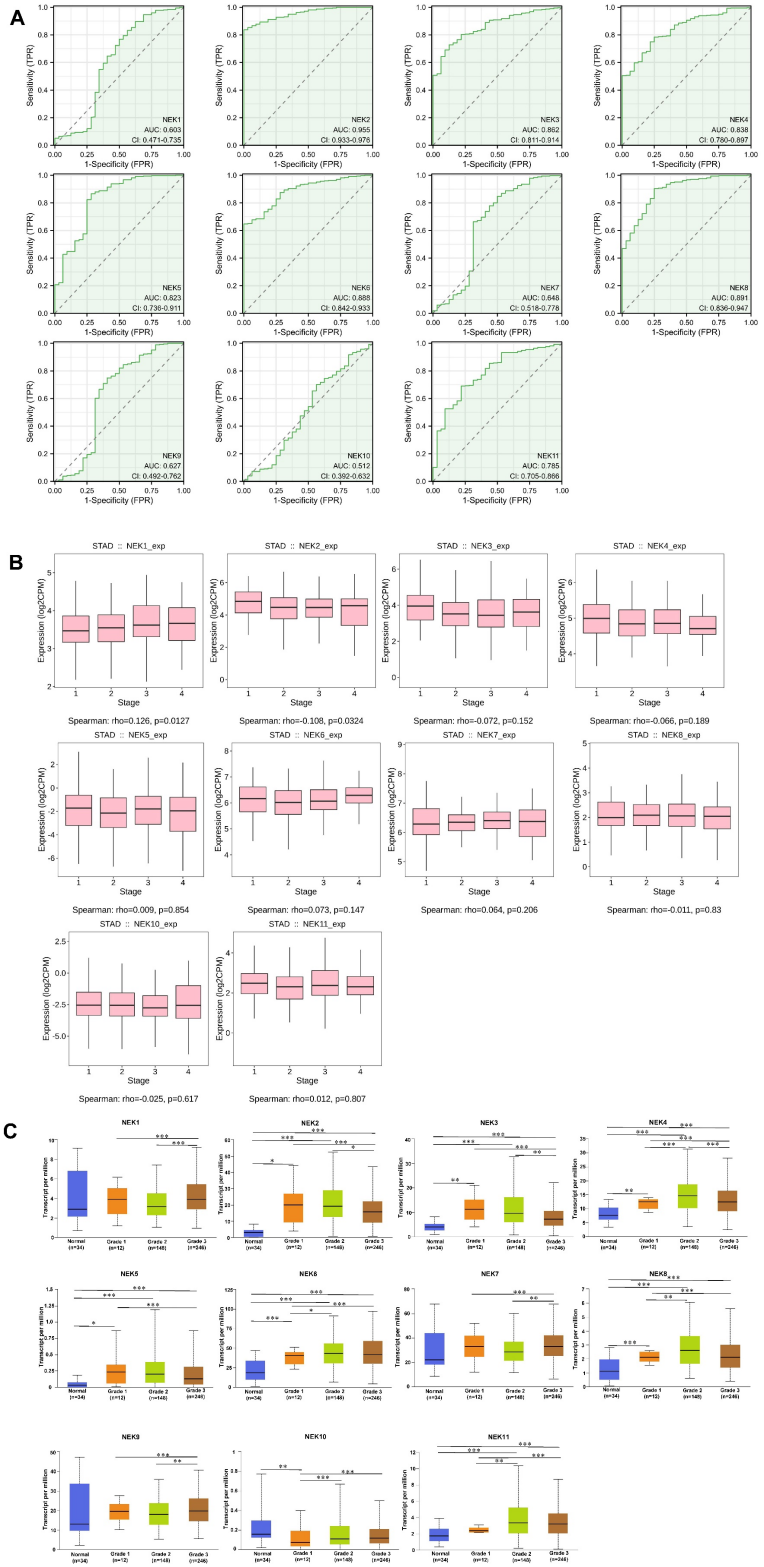
Association of NEK expression with STAD. (**A**) Diagnostic value of the NEK genes for STAD. (**B**) Correlations between the NEK expression levels and STAD stage. (**C**) Correlations between the NEK expression levels and STAD grade.

TCGA gastric cancer data from the Xiantao Academic Network were used to explore the diagnostic value of the NEK family members for STAD. As illustrated in **Figure 2A**, NEK2 (AUC = 0.955; 95% CI: 0.933-0.967) had excellent diagnostic capability, whereas NEK3 (AUC = 0.862; 95% CI: 0.811-0.914), NEK4 (AUC = 0.838; 95% CI: 0.780-0.897), NEK5 (AUC = 0.823; 95% CI: 0.736-0.911), NEK6 (AUC = 0.888; 95% CI: 0.842-0.933), and NEK8 (AUC = 0.891; 95% CI: 0.836-0.947) had moderate capabilities in this regard. By contrast, NEK1 (AUC = 0.603; 95% CI: 0.471-0.735), NEK7 (AUC = 0.648; 95% CI: 0.518-0.778), NEK9 (AUC = 0.627; 95% CI: 0.492-0.762), and NEK10 (AUC = 0.512; 95% CI: 0.392-0.632) had relatively poor diagnostic abilities. Overall, based on the results above, we conclude that the NEK family has relatively satisfactory potential for diagnosing STAD.

To gain better insight into the roles of the NEK family members in STAD development and progression, the TIDBS and UALCAN databases were used to further analyse the relationship between the gene expression levels and the clinicopathological characteristics of patients with the disease. As illustrated in **Figure 2B**, *NEK1* and *NEK2* expression was associated with the STAD stage (*P* < 0.05). However, there was no significant correlation between the expression of the other NEK genes and the cancer stage. Additionally, according to the UALCAN database, the mRNA expression levels of *NEK2, NEK3, NEK4, NEK5, NEK6,* and *NEK8* were significantly positively correlated with the tumour grade (**Figure 2C**), whereas that of *NEK10* was inversely associated with this factor. By contrast, *NEK1, NEK7,* and *NEK9* expression showed no obvious trend with the tumour grade.

Next, using TCGA data, we comprehensively analysed the correlation between *NEK* expression and clinicopathological parameters (**Table 2A, B**). The *NEK2* and *NEK4* expression levels were notably associated with the patient sex, whereas *NEK1* expression correlated strongly with T staging. Similarly, the NEK11 expression level correlated highly with the N and M stages of patients. In contrast to the UALCAN database results, the NEK6 and NEK7 expression levels significantly correlated with the patient grade, whereas *NEK1* expression correlated with the clinical stage.

### Prognostic value of the NEK family members for patients with STAD

**Figure 3:**
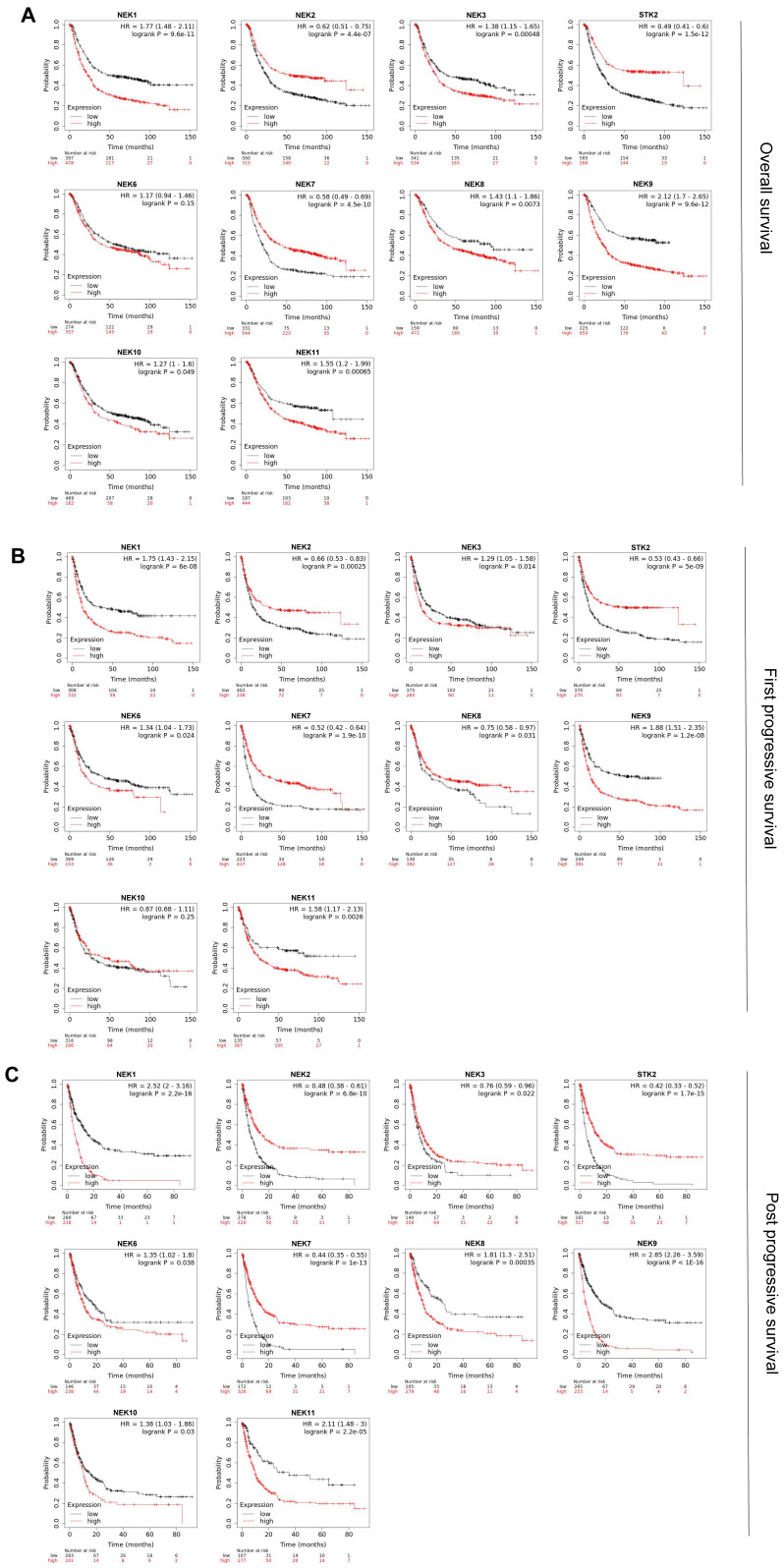
Prognostic value of the NEKs for patients with STAD. (**A**) Effects of the NEKs on the overall survival (OS) of the patients. (**B**) Effects of the NEKs on the first progressive survival (FP) of the patients. (**C**) Effects of the NEKs on the post-progressive survival (PPS) of the patients.

Kaplan-Meier analysis was used to investigate the prognostic value of the NEK family members for patients with STAD. **Figure 3A** illustrates the overall survival (OS) curve for the patients. In total 881 STAD samples in the Kaplan-Meier plotter were divided into high- and low-expression groups according to the mean values. Specifically, high expression of *NEK1, NEK3, NEK4, NEK8, NEK9, NEK10,* and *NEK11* correlated notably with poor OS. Conversely, high expression of *NEK2, NEK4,* and* NEK7* was notably associated with better OS, which is with the previous results showing their high differential expression in STAD. Subsequently, we explored the relationship between the NEKs and first progressive survival (FP) and post -progressive survival (PPS) of the patients. The results suggested that patients with higher levels of *NEK1, NEK3, NEK6, NEK9,* and *NEK11* had shorter FP, whereas those with higher levels of *NEK2, NEK4, NEK7,* and *NEK8* were associated with better FP (**Figure 3B**). Additionally, the upregulation of *NEK1, NEK6, NEK8, NEK9, NEK10,* and* NEK11* expression was related to poor PPS, whereas the overexpression of *NEK2, NEK3, NEK4,* and *NEK7* was linked to favourable PPS (**Figure 3C**). Taken together, these results indicate that the upregulation of *NEK1/3/9/11* and downregulation of *NEK2/4/7* expression are significantly associated with worse clinical outcomes in patients with STAD.

### *NEK* gene alterations in STAD

**Figure 4:**
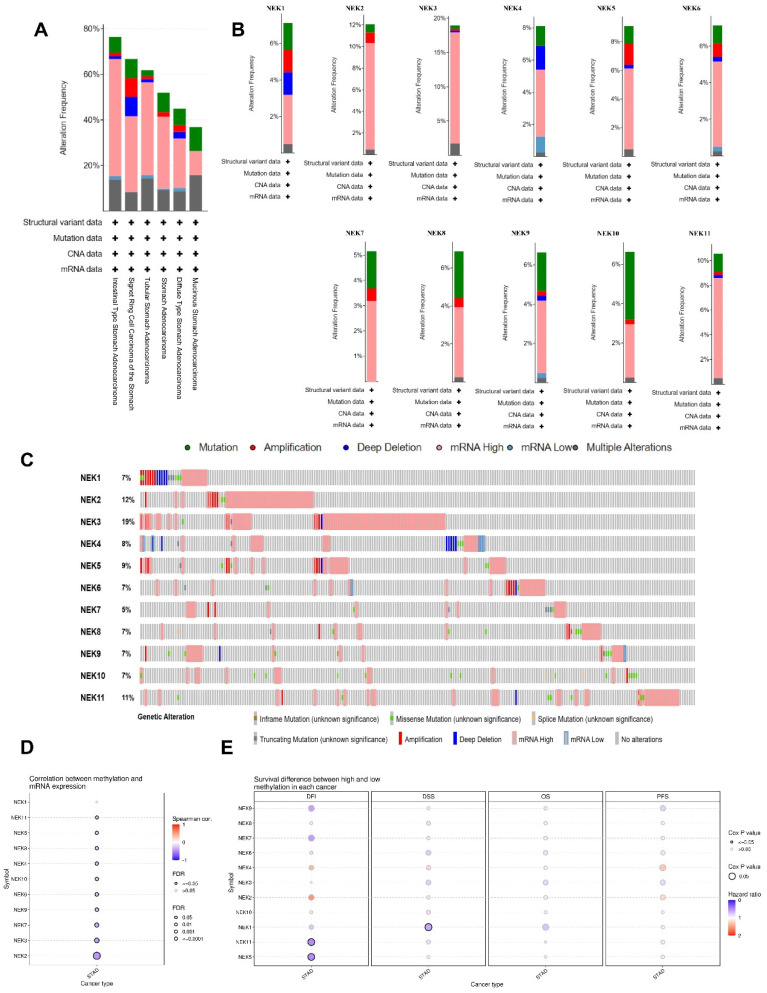
*NEK* gene alterations in STAD (cBioPortal). **(A)** *NEK* gene alterations in different gastric adenocarcinomas. **(B)** Genetic variations of the *NEK* family (*NEK1-NEK11*). **(C)** Summary of *NEK* gene alterations in STAD. **(D)** Correlation between NEK expression and methylation in STAD. **(E)** Survival differences between hypermethylated and hypomethylated patient groups.

Subsequently, we examined the genetic alterations in the NEK family in STAD using the cBioPortal database. In total, 233 (57%) of the 407 STAD samples showed genetic alterations, including missense mutations, amplifications, deep deletions, in-frame mutations, truncations, splice mutations, mRNA overexpression, and mRNA underexpression. mRNA overexpression was the dominant type of alteration in all STAD samples (**Figure 4A** and **B**). *NEK3, NEK2, NEK11, NEK9,* and *NEK8* were ranked as the top five genes with alterations, accounting for 19%, 12%, 11%, 9%, and 8% of the STAD samples, respectively (**Figure 4C**).

DNA methylation is an epigenetic mechanism which is closely related to the occurrence and development of tumours. We explored the NEK methylation levels in STAD using the GSCA database and found that the promoter methylation levels of all NEK family members, except NEK1, were decreased (**Figure 4D**). The *NEK2, NEK3,* and *NEK7* expression levels were moderately correlated with promoter methylation (r = -0.45, FDR = 0.00e+0; r = -0.40, FDR = 6.04e-16; r = -0.34, FDR = 3.73e-11, respectively). We also investigated the relationship between methylation expression and differences in patient survival, including disease-free interval (DFI), disease-specific survival (DSS), OS, and progression-free survival (PFS) (**Figure 4E**). Hypomethylation of *NEK5* and *NEK11* was significantly associated with worse DFI (Cox *P* = 0.035 and Cox *P* = 0.032, respectively), whereas hypomethylation of NEK1 was associated with poor DSS (Cox *P* = 0.024). One single CpG was shown to be prognostic for STAD (**Table 3**).

### Co-expression and functional analyses of the *NEK* genes in STAD

**Figure 5:**
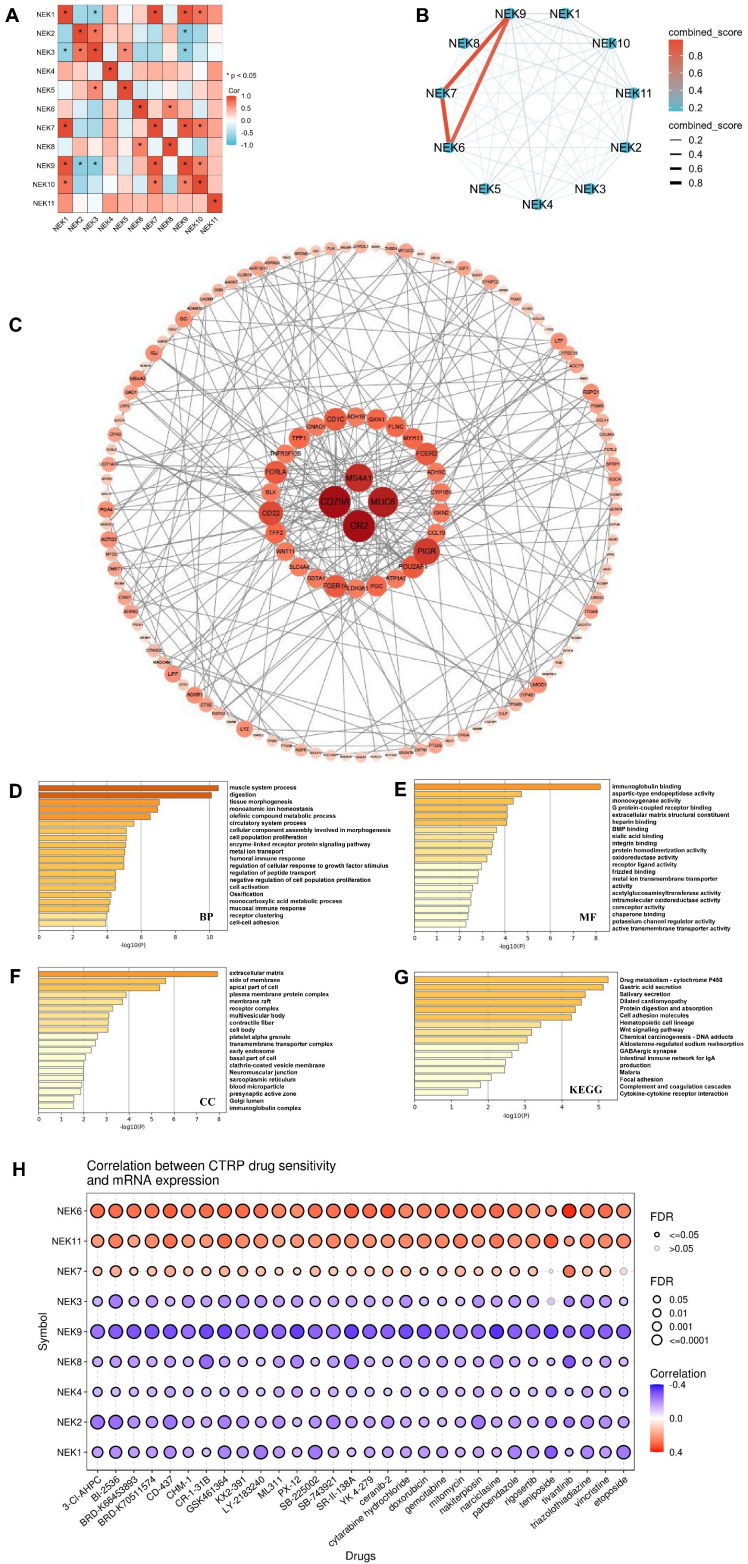
Interaction and functional analyses of NEKs in STAD. **(A)** Interaction analysis of the NEK family members (GEPIA). **(B)** Protein-protein interaction network of the NEK family in STAD (STRING).** (C)** Co-expression network of NEK family members in STAD. **(D)** Gene Ontology (GO) analysis of *N*EK-related biological processes (BP) in STAD. **(E)** GO analysis of *NEK*-related molecular functions (MF) in STAD. **(F)** GO analysis of *NEK*-related cellular components (CC) in STAD. **(G)** Kyoto Encyclopedia of Genes and Genomes (KEGG) analyses of NEK-related pathways in STAD. **(H)** Correlation between NEK expression and sensitivity to Cancer Therapeutics Response Portal (CTRP) drugs (top 30).

Using the GEPIA database, we analysed the relationships between the different *NEK* genes (**Figure 5A**). In the STAD group, a strong association was observed between *NEK1* and *NEK7/10* (*r* = 0.57, *P* < 0.001; *r* = 0.65, *P* < 0.001, respectively) as well as between *NEK7* and *NEK9* (*r* = 0.62, *P* < 0.001). *NEK3* was moderately related to *NEK2/5* (*r* = 0.25, *P* = 2.1e-07; *r* = 0.38, *P* = 3.6e-15, respectively). *NEK6* had a relatively moderate relationship with *NEK8* (*r* = 0.23, *P* = 81.7e-06). *NEK7* was moderately associated with NEK10 (r = 0.23, *P* = 3.8e-06). Next, we downloaded NEK-related genes (**Supplementary Table 2**) from the TCGA-STAD dataset of the cBioPortal database (cut-off: |Log_2_FC| > 1, *P* < 0.05) for co-expression and functional analyses. The protein-protein interaction network was plotted using the tools on the STRING and Cytoscape databases. **Figure 5B** illustrates the relationships between the different NEK proteins. As displayed in **Figure 5C**, B-cell antigen receptor complex-associated protein alpha chain isoform 1 (CD79a), mucin 4 subunit A1 (MUC4A1), mucin 6 (MUC6), and complement receptor type 2 (CR2) were the main molecules associated with the functional regulation of NEK family members in STAD.

To fully understand the downstream pathways of the NEKs in STAD, we used Metascape software to perform GO and KEGG enrichment analyses on 186 selected co-expressed genes to explore the potential biological functions of these kinases. As displayed in **Figure 5D**, the top-ranked biological processes related to the NEK genes were muscle system processes, digestion, tissue morphogenesis, monoatomic ion homeostasis, and olefinic component assembly involved in morphogenesis. Additionally, we found that humoral and mucosal immune responses were also important biological processes. With regard to cellular components, the extracellular matrix, side of the membrane, apical part of the cell, plasma membrane protein complex, membrane raft, and immunoglobulin complex were related to the NEK genes (**Figure 5E**). The most notable NEK gene-related molecular functions were immunoglobulin binding, aspartic-type endopeptidase activity, monooxygenase activity, G-protein-coupled receptor binding, and extracellular matrix structural constituents (**Figure 5F**). KEGG analysis indicated that the genes were enriched in pathways related to drug metabolism-cytochrome P450, gastric acid secretion, dilated cardiomyopathy, protein digestion and absorption, intestinal immune network for IgA production, and cytokine-cytokine receptor interactions (**Figure 5G**).

Given that the KEGG results had indicated a close association between the NEK family and drug metabolism in STAD, we used the Cancer Therapeutics Response Portal (CTRP) dataset from the GSCA database to predict the relationship between NEK expression and drug sensitivity. As displayed in **Figure 5H**, *NEK6* expression was positively correlated with sensitivity to BRD-K30748066 (*r* = 0.42, FDR = 0.008) and GSK-J4 (*r* = 0.40, FDR = 0.005). *NEK11* expression was positively correlated with sensitivity to BRD-K30748066 (*r* = 0.31, FDR = 0.056) and teniposide (*r* = 0.32, FDR = 2.54e-10). The expression of *NEK9* was negatively correlated with sensitivity to narciclasine (*r* =-0.35, FDR =1.15e-21), SR-II-138A (*r* =-0.33, FDR = 8.76e-21), tipifarnib-P2 (*r* =-0.32, FDR = 2.55e-10), teniposide (*r* =-0.32, FDR = 2.34e-10), and TG101348 (*r* = -0.32, FDR = 2.35e-17).

### Association between *NEK gene expression* and immune infiltration in STAD

**Figure 6:**
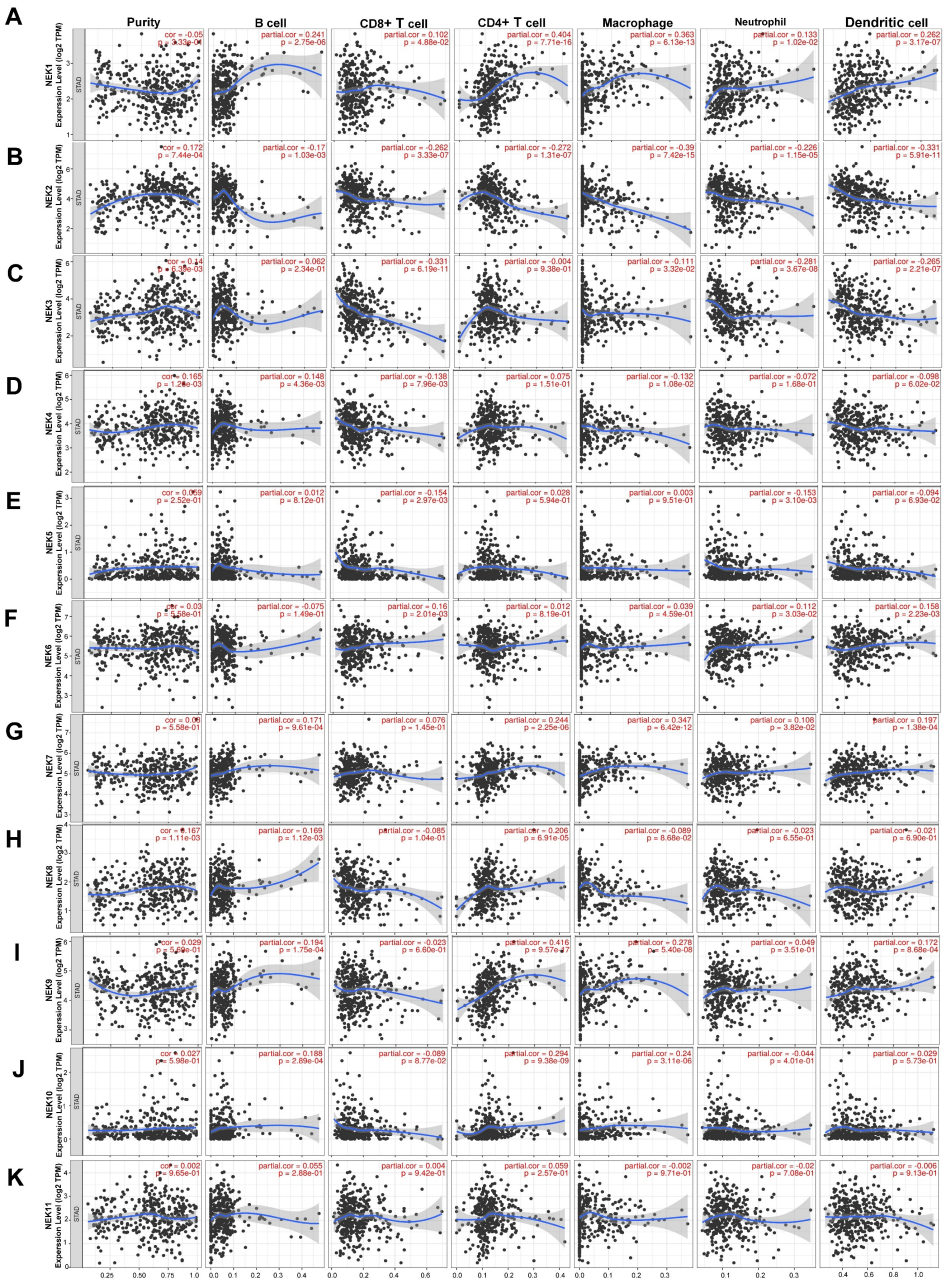
Association of *NEK* gene expression with immune infiltration in STAD. (**A**-**K**) Correlations of *NEK1-NEK11* expression levels with tumour immune infiltration (B cells, CD4+ T cells, CD8+ T cells, macrophages, neutrophils, and dendritic cells) in STAD.

In recent years, the immune microenvironment and immunotherapies have become relatively popular topics in cancer research 34, 35. Infiltrating immune cells constitute a significant portion of the tumour immune microenvironment, affecting the occurrence and development of tumours. Because NEK2 plays a pivotal role in the immune response of pancreatic cancer, NEK2 inhibitors can alleviate the immune resistance of this cancer type 36. Therefore, we first identified the relationship between each NEK family member and immune cell infiltration using resources on the TIMER database. As illustrated in **Figure 6,** *NEK1* expression was positively correlated with the infiltration of B cells, CD4+ T cells, CD8+ T cells, neutrophils, macrophages, and dendritic cells (DCs) (*P* < 0.05). By contrast, the *NEK2* expression level correlated negatively with the infiltration of these six types of immune cells (P < 0.05). *NEK3* expression was negatively associated with the infiltration of CD4+ T cells, CD8+ T cells, neutrophils, macrophages, and DCs (P < 0.05). *NEK4* expression correlated positively with B-cell infiltration but negatively with CD8+ T cells and macrophages (*P* < 0.05). *NEK5* expression correlated negatively with CD8+ T cells and neutrophil infiltration (*P* < 0.05). *NEK6* was positively associated with CD8+ T cells, neutrophils, and DCs (*P* < 0.05). The *NEK7* expression level correlated positively with the infiltration of all of the immune cells (*P* < 0.05), except for CD8+ T cells. *NEK8* expression was positively correlated with the infiltration of B and CD4+ T cells (*P* < 0.05). The *NEK9* expression level was positively correlated with the infiltration of all of the immune cells (*P* < 0.05), except for CD8+ T cells and neutrophils. *NEK10* expression was positively correlated with the infiltration of B cells, CD4+ T cells, and macrophages but negatively correlated with that of CD8+ T cells (*P* < 0.05). By contrast, there was no significant correlation between NEK11 and any of the six types of immune cells (*P* > 0.05).

### Association between *NEK* gene expression and immune cell markers

After analysing the associations of the NEK family members with tumour-infiltrating immune cells, we explored their correlations with immune cell markers. The results are summarised in** Table 4A** and **Table 4B**. The expression of *NEK1* was significantly correlated with most of the gene markers of B cells, T cells with different functions (CD8+ T, T-helper 1 (Th1), Th17, Th2, etc.), monocytes, DCs, neutrophils, and natural killer cells. *NEK2* expression correlated with markers of B cells, T cells, M2 macrophages, neutrophils, Th1 cells, monocytes, and DCs. *NEK3* expression was associated with B cell, CD8+ T cell, T cell, M2 macrophage, neutrophil, monocyte, and DC markers. *NEK4* was associated with Th17 cell and Treg markers. *NEK5* expression was associated only with markers of B cells. *NEK6* expression was correlated with markers of M2 macrophages, neutrophils, monocytes, Th17 cells, Tregs, and DCs. *NEK7* expression was associated with markers of T cells with different functions, M2 macrophages, neutrophils, and monocytes. *NEK8* expression was associated with Treg markers. *NEK9* expression was associated with markers of B cells, T cells with different functions, M2 macrophages, neutrophils, monocytes, and DCs. *NEK10* expression correlated with neutrophil markers. *NEK11* expression was associated with Tfh and Treg markers. These findings are all consistent with the results presented in **Figure 6K.**

### Relationship between *NEK* expression in STAD and tumour immune subtypes and immunotherapy

**Figure 7:**
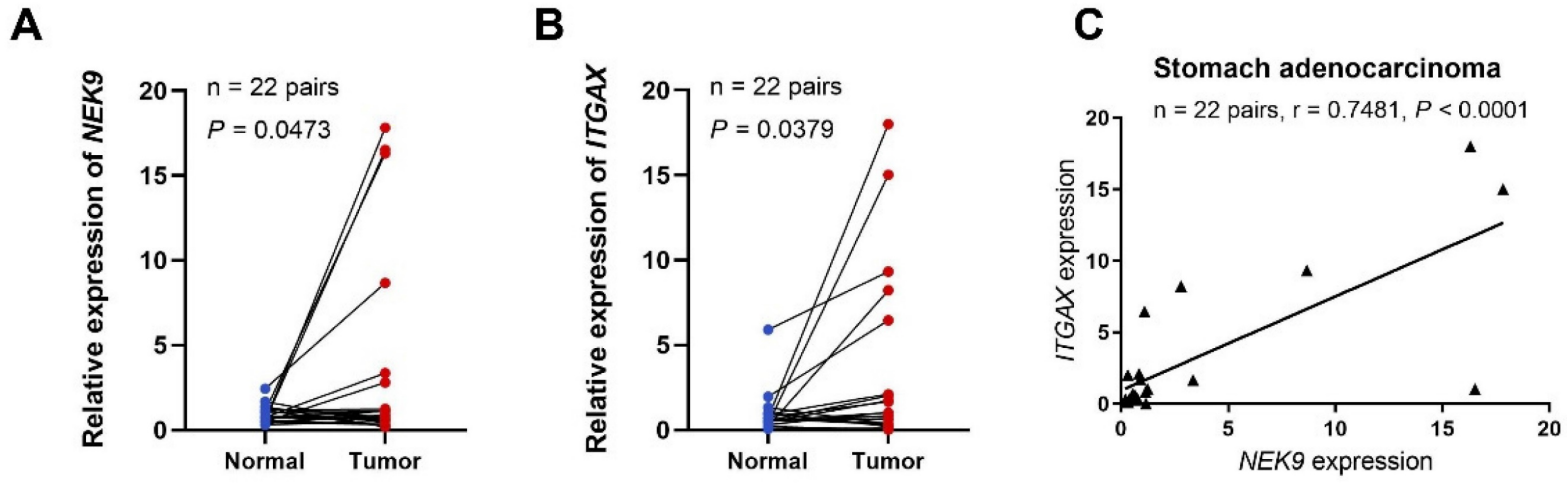
*NEK9* and *ITGAX* mRNA expression levels, and functional enrichment analysis of *NEK9* in STAD.** (A)** *NEK9* mRNA expression in STAD. **(B)** *ITGAX* mRNA expression in STAD. **(C)** Relationship between the mRNA expression levels of *NEK9* and* ITGAX* in STAD.

To further investigate the role of *NEK9* in STAD, we performed preliminary experiments to verify the differential expression of the gene in the tumour tissue and its correlation with immune cell markers. qRT-PCR analysis of *NEK9* expression in 22 STAD tissue samples revealed that the relative expression levels were significantly higher than those in adjacent non-tumour tissues (*P* = 0.0473; **Figure 7A**). We also demonstrated an association between NEK9 and integrin, alpha X (*ITGAX*), a marker gene for DCs 37. According to the qRT-PCR results, the relative expression levels of *ITGAX* in the 22 STAD samples were significantly higher than those in the matched normal samples taken from the vicinity of the cancerous tissue (*P* = 0.0379; **Figure 7**B). Finally, we found a positive correlation between the expression of *NEK9* and ITGAX in the 22 STAD samples (*r* = 0.7481, *P* < 0.001; **Figure 7C**).

**Figure 8:**
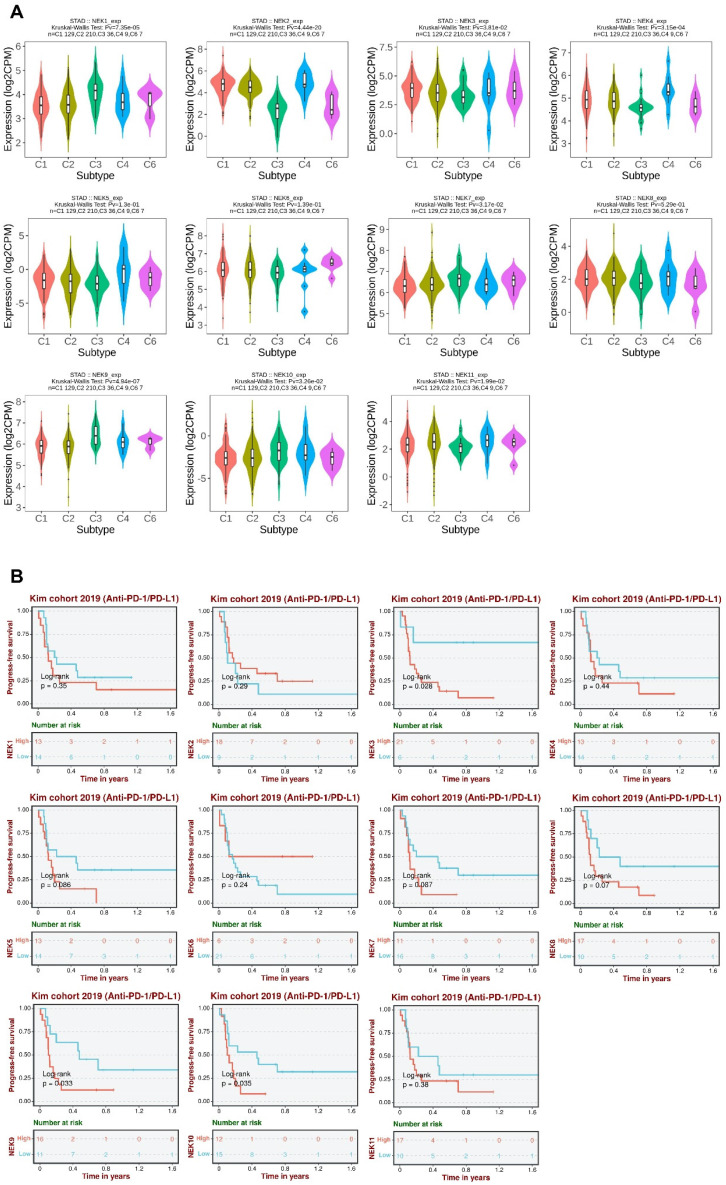
Association of NEKs with chemokines, chemokine receptors, and immune subtypes in STAD. **(A)** Relationships between the NEKs and immune subtypes in STAD (TISIDB). **(B)** Prognostic value of NEK expression levels after anti-PD1/PDL1 treatment (BEST).

To further elucidate the roles of NEKs in STAD pathogenesis, we investigated whether their expression differed amongst different immune subtypes of the disease (**Figure 8A**). The results indicated that the expression levels of *NEK1/2/3/4/7/9/10/11* differed significantly amongst the five immune subtypes (C1, C2, C3, C4, and C6). These findings further indicate that NEKs play a vital role in immune infiltration in STAD.

Next, we analysed the relationship between NEK expression and prognosis after anti-programmed death 1/programmed death ligand 1 (PD1/PDL1) treatment in patients with STAD. We found that high levels of *NEK3, NEK9,* and *NEK10* expression were associated with poor PFS after anti-PD1/PDL1 treatment (**Figure 8B**). These results suggest that members of the NEK family may be involved in resistance to STAD immunotherapy.

## Discussion

The NEK family consists of 11 protein kinases (*NEK1-NEK11*), which are primarily involved in checkpoint regulation, primary cilia function, mRNA splicing, and the cell cycle 38, 39. Because NEKs play an essential role in regulating the cell cycle and centrosome separation, their abnormal expression leads to chromosomal instability in tumour cells 40. Most NEK family members are differentially expressed in various types of cancer, such as those of the breast, lung, prostate, and colon-rectum 41-43. However, the carcinogenicity and association of NEKs with STAD have not been fully elucidated. Therefore, it is necessary to comprehensively investigate the expression profiles of the NEKs in STAD and their association with immune cell infiltration as well as their prognostic value.

Previous studies have revealed the abnormal expression of NEK family members in a variety of tumours. Their expression levels correlate with the clinical characteristics and prognosis of cancer patients and can be used as diagnostic and prognostic markers. Zhu et al. 44 demonstrated that *NEK1* expression was elevated in glioma tissues and cells compared with the level in normal brain tissues, and its high level was associated with expression of the tumour cell proliferation marker Ki-67, the tumour grade, and poor survival. *NEK2* is upregulated in hepatocellular carcinoma (HCC) and is associated with adverse outcomes in patients with this disease 45. In STAD, NEK2 has been shown to play a cancer-promoting role by activating the AKT-mediated signaling pathway 46. Fang et al. showed that MBM-5 can effectively inhibit the kinase activity of NEK2, which has potential application value in the anti-gastric cancer and colorectal cancer 47.

*NEK3* overexpression is significantly correlated with the TNM stage, lymph node metastasis, and poor prognosis of patients with gastric cancer and can be used as an independent prognostic factor of patient survival 48. According to Ding et al. 12, *NEK4* overexpression promotes the migration and invasion of lung cancer cells and is a promising diagnostic marker of lung cancer metastasis. *NEK6* is an adverse prognostic factor in HCC, and its expression correlates with the histological grade, Ki-67 expression, and alpha-foetal protein level 49. A study on *NEK7* evidenced that its expression was upregulated and associated with a poor prognosis in STAD 50. In gastric cancer, NEK8 has been confirmed to indirectly affect the survival rate of gastric cancer patients through its direct interaction with von-Hippel-Lindau tumor suppressor protein (pVHL) 51. In our study, the *NEK1/2/3/4/5/6/7/8/9/11* expression levels were substantially higher in patients with STAD. This result is consistent with those of other studies on the expression of NEKs, such as* NEK3* and *NEK7* in this disease. Furthermore, our study found that high *NEK1/6/7/9/11* expression was closely associated with clinicopathological parameters such as the TMN stages and clinical grade of patients with STAD. In terms of diagnosis and prognosis, *NEK2/3/4/5/6/8* overexpression has good diagnostic value in STAD. High* NEK1/8/9/10/11* expression was correlated with poor OS and PPS, whereas high *NEK1/6/9/11* expression was associated with poor FP. These results highlight the potential use of the NEK family members as diagnostic and prognostic markers for STAD.

We also investigated NEK gene alterations in STAD tissue specimens from patients at the Xiangya Hospital. Of the 407 STAD samples examined, 233 (57%) showed genetic alterations, including missense mutations, amplifications, deep deletions, in-frame mutations, truncations, splicing mutations, mRNA overexpression, and mRNA underexpression. *NEK3, NEK2, NEK11, NEK9,* and *NEK8* were the five genes with the most alterations, accounting for 19%, 12%, 11%, 9%, and 8% of the STAD samples, respectively. DNA methylation is an epigenetic mechanism that is closely associated with the occurrence and development of tumours. Using the GSCA database, we found that the promoter methylation levels of all the NEK genes, except NEK1, were downregulated in STAD. We also investigated the relationship between methylation status and patient survival and found that the low methylation of NEK5 and NEK11 was significantly associated with poor DFI, and the low methylation of NEK1 was associated with poor DSS.

According to the GO and KEGG enrichment analyses, the NEKs are closely associated with immune function, being involved in the humoral and mucosal immune responses, immunoglobulin complex, immunoglobulin binding, intestinal immune network for IgA production, and cytokine-cytokine receptor interactions. Recent studies have evidenced that immune cell infiltration and the tumour microenvironment are involved in the progression and immune escape of STAD 52-55. In this study, the correlation between the NEKs and immune infiltration was analysed using the TIMER and TISIDB databases. The results revealed that the NEKs were highly associated with the activity and expression of numerous immune cells, immunoregulators and their receptors, and immune subtypes. Specifically, the expression of *NEK2/3/4/5/6* was highly correlated with CD8+ T cell infiltration, whereas that of *NEK1/2/3/6/9* was associated with DCs. Moreover, the *NEK1/4/6/7/8/9/11* expression levels were associated with Treg infiltration. CD8+ T cells, natural killer cells, and DCs play extremely important roles in anti-tumour immunotherapy, whereas Tregs play a cancer-promoting role in the tumour immune microenvironment 56. Therefore, we hypothesise that *NEK1/4/6/7/8/9/11* may play a cancer-promoting role in STAD via Tregs. Moreover, as the most effective antigen-presenting cells, activated DCs can stimulate the anti-tumour immune response of T and NK cells 57. However, there are no published reports on the association of* NEK9* with DCs in STAD. Therefore, using 22 pairs of FFPE archival STAD tissue and matched adjacent normal tissue samples, we further confirmed the differential expression of *NEK9* in STAD and its correlation with immune cells and verified that the gene expression level was markedly upregulated in the cancerous tissue. *NEK9* also evidenced a positive correlation with *ITGAX*, which is a marker gene of DCs. These results suggest that NEK9 may have anti-STAD effects through its interaction with DCs. *CXCR4*, a representative G-protein-coupled receptor, plays an important role in mediating tumour-directed migration, invasion, and metastasis. These findings suggest that the impact of the NEKs on the prognosis of patients with STAD may be partly explained by their regulation of the tumour immune microenvironment. This hypothesis warrants further research. Additionally, our study suggests that *NEK9* plays a major role in tumour immunity and is therefore a potential biomarker for predicting both the disease prognosis and the efficacy of immunotherapy in patients with STAD.

As a limitation of this study, the relationships between the *NEK* family members and STAD were explored through bioinformatic analysis and lacked experimental clinical validation. Hence, more studies will be conducted to analyse and confirm the specific mechanisms of action of these NEKs in order to support their clinical application as prognostic biomarkers or immunotherapeutic targets for STAD.

In conclusion, we investigated the roles of NEKs in the pathogenesis of STAD by analysing their differential expression, gene mutations, functional enrichment, and associations with immune infiltration and patient prognosis. Our findings suggest promising new directions for the research and development of STAD treatments.

## Supplementary Material

Supplementary table 1.

Supplementary table 2.

## Figures and Tables

**Table 1:** Primer sequences used for the qRT-PCR.

Gene	Primer (Forward)	Primer (Reverse)
*NEK9*	GTGGAAGGAAGTCGATTTGACC	GCAGTGCCAGAATAACAATCTCA
*ITGAX*	GGGATGCCGCCAAAATTCTC	ATTGCATAGCGGATGATGCCT
*U6*	CTCGCTTCGGCAGCACA	AACGCTTCACGAATTTGCGT

**Table 2A:** Clinicopathologic parameters and the expressions of NEK family members in STAD.

Characteristics	*N*	NEK1			NEK2			NEK3			NEK4			NEK5			NEK6		
		High	Low	*P*	High	Low	*P*	High	Low	*P*	High	Low	*P*	High	Low	*P*	High	Low	*P*
**Gender**				0.775			0.001			0.512			0.002			0.650			0.625
Male	221	86	135		77	144		73	148		75	146		79	142		94	127	
Female	126	51	75		66	60		46	80		64	62		42	84		59	69	
Age				0.545			0.432			0.916			0.804			0.918			0.363
≤60	115	48	67		44	71		39	76		45	70		40	75		54	61	
>60	232	89	143		99	133		80	152		94	138		81	151		97	135	
**T Stage**				0.028			0.980			0.819			0.906			0.620			0.233
T_1_+T_2_+T_3_	250	88	162		103	147		88	162		101	149		85	165		111	139	
T_4_	97	49	48		40	57		31	66		38	59		36	61		40	57	
**N Stage**				0.075			0.653			0.715			0.096			0.976			0.858
Nx+N_0_+N_1_+N_2_	275	102	173		115	160		182	93		104	171		96	179		119	156	
N_3_	72	35	37		28	44		26	46		35	37		25	47		32	40	
**Stage**				0.002			0.639			0.251			0.361			0.341			0.352
StageⅠ+Ⅱ	162	53	109		69	93		50	112		67	95		50	112		65	97	
Stage Ⅲ	148	71	77		58	90		58	90		54	94		57	91		71	77	
Stage Ⅳ	37	13	24		16	21		11	26		18	19		14	23		15	22	
**Grade**				1.000			1.000			0.315			0.090			0.424			0.039
G_1_	8	3	5		3	5		4	4		6	2		2	6		7	1	
G_2_+G_3_	331	131	200		137	194		114	217		200	131		118	213		140	191	
G_x_	8	3	5		3	5		1	7		2	6		1	7		4	4	
**M Stage**				0.784			0.961			0.918			0.110			0.779			0.812
M_0_+ M_x_	323	130	193		131	192		111	212		126	197		112	211		140	183	
M_1_	24	7	17		12	12		8	16		13	11		9	15		11	13	

**Table 2B:** Clinicopathologic parameters and the expressions of NEK family members in STAD (continued).

Characteristics	*N*	NEK7			NEK8			NEK9			NEK10			NEK11		
		High	Low	*P*	High	Low	*P*	High	Low	*P*	High	Low	*P*	High	Low	*P*
**Gender**				0.542			0.380			0.116			0.513			0.199
Male	221	88	133		91	130		107	114		76	145		81	140	
Female	126	46	80		58	68		76	50		39	87		55	71	
Age				0.400			0.215			0.363			0.319			0.166
≤60	115	48	67		44	71		59	56		34	81		51	64	
>60	232	86	146		105	127		131	101		81	151		85	147	
**T Stage**				0.646			0.676			0.314			0.672			0.112
T_1_+T_2_+T_3_	250	93	157		110	140		119	131		80	170		91	159	
T_4_	97	41	56		39	58		38	59		35	62		45	52	
**N Stage**				0.745			0.435			0.675			0.809			0.106
Nx+N_0_+N_1_+N_2_	275	105	170		121	154		126	149		92	183		175	100	
N_3_	72	29	43		28	44		31	41		23	49		36	36	
**Stage**				0.458			0.338			0.865			0.561			0.137
StageⅠ+Ⅱ	162	57	105		74	88		74	88		49	113		59	103	
Stage Ⅲ	148	61	87		57	91		65	83		53	95		57	91	
Stage Ⅳ	37	16	21		18	19		18	19		13	24		20	17	
**Grade**				0.006			0.927			0.043			0.071			0.790
G_1_	8	6	2		4	4		6	2		4	4		3	5	
G_2_+G_3_	331	128	203		142	189		150	181		111	220		131	200	
G_x_	8	0	8		3	5		1	7		0	8		2	6	
**M Stage**				0.750			0.249			0.952			0.983			0.044
M_0_+ M_x_	323	124	199		132	187		146	177		107	216		122	201	
M_1_	24	10	14		13	11		11	13		8	16		14	10	

**Table 3:** The prognostic values of CpG sites in the NEKs by MethSurv**.**

CpG site	Gene symbol	Group	CpG Island	HR	CI	*P* value
cg02998883	NEK1	Body	Open_Sea	0.649	0.45-0.935	0.017
cg05110629	NEK1	Body	Open_Sea	0.664	0.452-0.974	0.03
cg26722769	NEK1	Body	Open_Sea	0.651	0.434-0.977	0.031
cg11225435	NEK2	TSS200	Island	0.668	0.481-0.928	0.018
cg15731669	NEK3	5'UTR;TSS200;Body	Island	0.701	0.498-0.986	0.046
cg22900224	NEK4	5'UTR;1stExon	Island	0.688	0.491-0.964	0.027
cg14049380	NEK4	TSS200	Island	0.68	0.491-0.942	0.019
cg03143060	NEK4	TSS1500	S_Shore	0.498	0.36-0.688	2.20E-05
cg08090396	NEK4	TSS1500	S_Shore	0.699	0.487-1.002	0.046
cg00883505	NEK5	TSS200	Island	0.708	0.512-0.979	0.036
cg18057513	NEK5	TSS1500	S_Shore	0.54	0.373-0.781	0.00061
cg20559216	NEK5	TSS1500	S_Shore	0.643	0.447-0.924	0.014
cg08287471	NEK6	5'UTR;Body	Island	0.645	0.467-0.892	0.0088
cg14196208	NEK6	TS200;TSS1500;Body	N_Shore	0.707	0.512-0.977	0.035
cg13582060	NEK6	5'UTR;Body	Open_Sea	0.711	0.511-0.988	0.04
cg14036069	NEK6	5'UTR;Body	Open_Sea	0.711	0.516-0.98	0.037
cg08528000	NEK7	Body	Open_Sea	0.536	0.379-0.76	0.00029
cg12750917	NEK7	Body	Open_Sea	0.702	0.51-0.968	0.031
cg14557909	NEK8	TSS1500	Island	0.568	0.361-0.895	0.0093
cg05343811	NEK9	Body	S_Shore	1.74	1.186-2.551	0.003
cg17147885	NEK10	5'UTR	Open_Sea	0.521	0.343-0.791	0.0011
cg09642369	NEK10	TSS1500	S_Shore	0.572	0.412-0.795	0.0011
cg17918906	NEK10	TSS1500	S_Shore	0.558	0.398-0.782	0.0011
cg20886017	NEK11	Body	Open_Sea	1.468	1.064-2.025	0.02

**Table 4A:** The correlations between the expression of NEK family members and the markers of immune cells.

		NEK1		NEK2		NEK3		NEK4		NEK5		NEK6	
		Cor	*P*	Cor	*P*	Cor	*P*	Cor	*P*	Cor	*P*	Cor	*P*
CD8+ Tcell	CD8A	0.315	***	-0.191	***	-0.285	***	0.049	0.320	-0.111	*	0.123	*
	CD8B	0.179	***	-0.053	0.278	-0.101	*	0.072	0.141	-0.074	0.130	0.037	0.449
	GZMA	0.225	***	-0.081	0.099	-0.216	***	0.032	0.518	-0.116	*	0.085	0.083
B cell	CD19	0.276	***	-0.224	***	-0.190	***	0.002	0.968	-0.112	**	0.064	0.195
	CD79A	0.180	***	-0.337	***	-0.281	***	-0.100	*	-0.164	*	0.082	0.094
	MS4A1	0.324	***	-0.366	***	-0.219	***	-0.056	0.257	-0.105	*	0.072	0.146
T cell	CD3D	0.218	***	-0.222	***	-0.261	***	-0.054	0.272	-0.163	**	0.114	*
	CD3E	0.239	***	-0.249	***	-0.251	***	-0.033	0.502	-0.130	**	0.135	**
	CD2	0.303	***	-0.193	***	-0.201	***	0.031	0.535	-0.088	0.073	0.136	**
M2	MS4A4A	0.390	***	-0.248	***	-0.179	***	0.018	0.716	0.020	0.682	0.187	***
	CD163	0.426	***	-0.122	*	-0.130	**	0.168	**	0.086	0.082	0.290	***
	VSIG4	0.312	***	-0.195	***	-0.179	***	0.016	0.746	0.027	0.579	0.173	***
Neutrophils	ITGAM	0.379	***	-0.193	***	-0.184	***	0.069	0.160	0.018	0.709	0.308	***
	CCR7	0.335	***	-0.343	***	-0.226	***	-0.032	0.516	-0.059	0.227	0.205	***
	SIGLEC5	0.378	***	-0.197	***	-0.112	*	0.100	*	0.040	0.413	0.173	***
DC	ITGAX	0.334	***	-0.152	**	-0.102	*	0.160	**	0.031	0.528	0.295	***
	CD1C	0.324	***	-0.440	***	-0.209	***	-0.116	**	-0.085	0.085	0.101	*
	NRP1	0.460	***	-0.235	***	-0.113	*	0.077	0.116	0.068	0.169	0.282	***
NK cell	KIR3DL3	0.023	0.644	0.089	0.070	-0.047	0.342	0.086	0.080	0.049	0.324	0.036	0.469
	KIR2DS4	0.158	**	-0.037	0.458	-0.108	*	0.044	0.368	0.000	0.994	-0.034	0.488
Th1	TBX21	0.297	***	-0.159	**	-0.246	***	0.077	0.119	-0.077	0.115	0.197	***
	STAT1	0.270	***	0.267	***	0.004	0.936	0.364	***	0.123	*	0.184	***
	STAT4	0.439	***	-0.202	***	-0.105	*	0.100	*	0.009	0.850	0.157	**
	IFNG	0.171	***	0.117	*	-0.077	0.119	0.151	**	-0.035	0.478	0.096	0.052
Th2	STAT6	0.269	***	-0.043	0.378	0.047	0.335	0.180	***	0.156	**	0.303	***
	GATA3	0.257	***	-0.252	***	-0.304	***	-0.073	0.139	-0.105	*	0.077	0.117
	STAT5A	0.476	***	-0.093	0.059	-0.047	0.337	0.232	***	0.108	*	0.279	***
Tfh	BCL6	0.481	***	-0.327	0.000	-0.230	***	0.133	**	0.048	0.334	0.209	***
	IL21	0.209	***	0.063	0.200	-0.069	0.158	0.095	0.053	0.056	0.253	0.074	0.134
Th17	STAT3	0.502	***	-0.053	0.282	-0.069	0.158	0.384	***	0.199	***	0.401	***
	IL17A	-0.102	*	0.165	0.001	0.096	0.051	0.137	**	0.004	0.937	0.152	**
Treg	FOXP3	0.254	***	-0.045	0.356	-0.095	0.054	0.136	**	-0.031	0.530	0.217	***
	STAT5B	0.614	***	-0.107	0.029	0.090	0.066	0.306	***	0.213	***	0.286	***
	CCR8	0.343	***	-0.035	0.473	-0.076	0.122	0.196	***	0.047	0.340	0.223	***
T exhaustion-cell	PDCD1	0.229	***	-0.052	0.293	-0.209	***	0.069	0.163	-0.086	0.081	0.184	***
	CTLA4	0.277	***	0.046	0.354	-0.021	0.671	0.208	***	0.023	0.645	0.134	**
	HAVCR2	0.321	***	-0.107	0.029	-0.167	**	0.091	0.063	0.018	0.716	0.228	***
	LAG3	0.188	***	-0.029	0.553	-0.260	***	0.050	0.310	-0.121	*	0.094	0.057
Monocyte	CD86	0.292	***	-0.156	0.001	-0.174	***	0.039	0.422	0.000	0.998	0.174	***
	C3AR1	0.368	***	-0.180	0.000	-0.148	**	0.061	0.216	0.053	0.277	0.227	***
	CSF1R	0.395	***	-0.239	0.000	-0.202	***	0.077	0.119	0.024	0.625	0.258	***

**Table 4B:** The correlations between the expression of NEK family members and the markers of immune cells (continued).

		NEK7		NEK8		NEK9		NEK10		NEK11			
		Cor	*P*	Cor	*P*	Cor	*P*	Cor	*P*	Cor	*P*		
CD8+ Tcell	CD8A	0.187	***	-0.018	0.716	0.183	***	-0.014	0.782	0.086	0.082		
	CD8B	0.099	*	0.028	0.569	0.066	0.183	0.054	0.272	0.040	0.418		
	GZMA	0.119	*	-0.115	*	0.104	*	-0.099	*	0.058	0.234		
B cell	CD19	0.078	0.114	0.122	*	0.222	***	0.061	0.218	-0.008	0.877		
	CD79A	0.038	0.446	0.037	0.458	0.097	*	0.018	0.709	-0.074	0.132		
	MS4A1	0.149	**	0.034	0.485	0.233	***	0.076	0.124	-0.049	0.324		
T cell	CD3D	0.090	0.067	-0.064	0.195	0.107	*	-0.054	0.274	-0.011	0.828		
	CD3E	0.084	0.087	0.003	0.945	0.141	**	0.003	0.957	0.049	0.319		
	CD2	0.169	**	-0.020	0.682	0.199	***	0.024	0.621	0.067	0.174		
M2	MS4A4A	0.327	***	-0.075	0.126	0.281	***	0.114	*	0.057	0.251		
	CD163	0.374	***	0.028	0.574	0.368	***	0.158	**	0.165	**		
	VSIG4	0.265	***	-0.107	*	0.227	***	0.059	0.227	0.069	0.158		
Neutrophils	ITGAM	0.322	***	0.142	**	0.323	***	0.169	**	0.153	**		
	CCR7	0.184	***	0.084	0.088	0.262	***	0.127	*	0.010	0.842		
	SIGLEC5	0.280	***	0.025	0.613	0.308	***	0.121	*	0.030	0.549		
DC	ITGAX	0.277	***	0.149	**	0.305	***	0.121	*	0.110	*		
	CD1C	0.202	***	0.061	0.218	0.241	***	0.178	***	-0.026	0.590		
	NRP1	0.428	***	-0.001	0.986	0.439	***	0.269	***	0.148	**		
NK cell	KIR3DL3	0.014	0.777	0.028	0.571	-0.037	0.450	0.008	0.869	0.075	0.127		
	KIR2DS4	0.094	0.056	-0.058	0.237	0.084	0.087	-0.010	0.837	0.053	0.285		
Th1	TBX21	0.131	**	0.046	0.346	0.210	***	-0.012	0.811	0.088	0.074		
	STAT1	0.300	***	0.163	**	0.264	***	0.066	0.178	0.359	***		
	STAT4	0.320	***	0.068	0.167	0.352	***	0.175	***	0.144	**		
	IFNG	0.116	*	-0.024	0.628	0.063	0.200	-0.051	0.298	0.132	**		
Th2	STAT6	0.350	***	0.317	***	0.428	***	0.192	***	0.289	***		
	GATA3	0.175	***	0.059	0.232	0.173	***	0.027	0.577	0.051	0.298		
	STAT5A	0.404	***	0.207	***	0.458	***	0.173	***	0.238	***		
Tfh	BCL6	0.345	***	0.088	0.072	0.393	***	0.269	***	0.167	**		
	IL21	0.143	**	0.035	0.471	0.106	*	0.044	0.371	0.115	*		
Th17	STAT3	0.414	***	0.308	***	0.531	***	0.318	***	0.390	***		
	IL17A	-0.193	***	0.014	0.781	-0.119	*	0.046	0.346	0.019	0.700		
Treg	FOXP3	0.098	*	0.190	***	0.186	***	0.060	0.219	0.159	**		
	STAT5B	0.530	***	0.318	***	0.693	***	0.389	***	0.234	***		
	CCR8	0.266	***	0.151	**	0.299	***	0.178	***	0.224	***		
T exhaustion-cell	PDCD1	0.085	0.082	0.113	*	0.183	***	-0.064	0.194	0.087	0.077		
	CTLA4	0.132	0.007	0.081	0.100	0.190	***	0.062	0.207	0.168	**		
	HAVCR2	0.263	***	-0.008	0.864	0.235	***	0.023	0.643	0.119	*		
Monocyte	CD86	0.241	***	-0.051	0.304	0.189	***	0.046	0.347	0.066	0.181		
	C3AR1	0.319	***	-0.039	0.423	0.298	***	0.093	0.058	0.063	0.198		
	CSF1R	0.295	***	0.065	0.186	0.338	***	0.152	**	0.081	0.101		

* p < 0.05; ** p < 0.01; *** p < 0.001
